# Competitive Li^+^ Coordination in Ionogel Electrolytes for Enhanced Li‐Ion Transport Kinetics

**DOI:** 10.1002/advs.202300226

**Published:** 2023-06-06

**Authors:** Jiafeng Li, Tao Zhang, Xiaobin Hui, Ruixiao Zhu, Qiqi Sun, Xiaoxuan Li, Longwei Yin

**Affiliations:** ^1^ Key Laboratory for Liquid‐Solid Structural Evolution and Processing of Materials Ministry of Education School of Materials Science and Engineering Shandong University Jinan 250061 P. R. China

**Keywords:** ionic conductivity, ionogel electrolytes, Li metal batteries, Li^+^ transport kinetics, zwitterion

## Abstract

Developing ionogel electrolytes based on ionic liquid instead of volatile liquid in gel polymer electrolytes is regarded to be effective to diminish safety concerns in terms of overheating and fire. Herein, a zwitterion‐based copolymer matrix based on the copolymerization of trimethylolpropane ethoxylate triacrylate (ETPTA) and 2‐methacryloyloxyethylphosphorylcholine (MPC, one typical zwitterion) is developed. It is shown that introducing zwitterions into ionogel electrolytes can effectively optimize local lithium‐ion (Li^+^) coordination environment to improve Li^+^ transport kinetics. The interactions between Li^+^ and bis(trifluoromethanesulfonyl)imide (TFSI^−^)/MPC lead to the formation of Li^+^ coordination shell jointly occupied by MPC and TFSI^−^. Benefiting from the competitive Li^+^ attraction of TFSI^−^ and MPC, the energy barrier of Li^+^ desolvation is sharply decreased and thus the room‐temperature ionic conductivity can reach a value of 4.4 × 10^−4^ S cm^−1^. Besides, the coulombic interaction between TFSI^−^ and MPC can greatly decrease the reduction stability of TFSI^−^, boosting in situ derivation of LiF‐enriched solid electrolyte interface  layer on lithium metal surface. As expected, the assembled Li||LiFePO_4_ cells deliver a high reversible discharge capacity of 139 mAh g^−1^ at 0.5 C and good cycling stability. Besides, the pouch cells exhibit a steady open‐circuit voltage and can operate normally under abuse testing (fold, cut), showing its outstanding safety performance.

## Graphical abstract

The similar lithium‐ion (Li^+^) binding energies of the 2‐methacryloyloxyethylphosphorylcholine (MPC) and bis(trifluoromethanesulfonyl)imide (TFSI^−^) lead to the formation of Li^+^ coordination shell jointly occupied by MPC and TFSI^−^. Benefiting from the competitive Li^+^ attraction of TFSI^−^ and MPC, the energy barrier of Li^+^ desolvation sharply decreases, leading to improved ion transport kinetics.

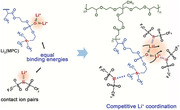

## 1 Introduction

Lithium (Li) metal has been considered as a promising anode for next‐generation lithium‐based batteries owing to its extremely high theoretical capacity (3860 mAh g^−1^) and the lowest electrochemical potential (−3.04 V vs the standard hydrogen electrode).^[^ ^1^, ^2^, ^3^ ^]^ However, irreversible parasitic reactions between liquid electrolytes and lithium metal would lead to premature battery failure. The uncontrolled lithium dendrites also limit both the performance and safety even at current densities below the diffusion limiting current.^[^ ^4^, ^5^ ^]^ To alleviate these issues related to lithium metal batteries, some possible approaches have been demonstrated effective to address these challenges, including chemical modification of anode surface,^[^ ^6^, ^7^, ^8^, ^9^ ^]^ electrolyte engineering,^[^ ^10^, ^11^, ^12^ ^]^ or developing solid‐state electrolytes.^[^ ^13^, ^14^, ^15^ ^]^ Therein, solid‐state electrolytes featuring inherent safety properties, wide operating voltage (>4.5 V vs Li/Li^+^) and capacity to suppress dendrite growth are considered particularly desirable to revive high‐energy‐density Li‐based batteries.^[^ ^16^, ^17^ ^]^

Among all solid‐state electrolytes, solid polymer electrolytes consisting of organic polymer and lithium salt are attractive by virtue of low cost, lightweight, superior flexibility, and facile preparation.^[^ ^18^ ^]^ In general, a satisfactory solid polymer electrolyte is required to possess fast bulk/interfacial ion transport and remain excellent chemical stability during long‐tern battery cycling. However, for the common solid polymer electrolytes, Li^+^ transport only relies on sluggish segmental motion of amorphous region, could not meet high requirements on fast charge transfer kinetics for high electrochemical energy storage devices.^[^ ^19^ ^]^ Several strategies, such as cross‐linking,^[^ ^20^, ^21^, ^22^ ^]^ copolymerization,^[^ ^23^, ^24^ ^]^ and introducing inorganic fillers,^[^ ^25^, ^26^, ^27^, ^28^, ^29^ ^]^ have been put forward to increase local relaxation ability of polymer chain. However, the sluggish lithium ion transport kinetics cannot be fundamentally enhanced based on the present methods.

Gel polymer electrolytes (GPEs) composed of polymeric scaffold and a high‐volume‐fraction plasticizer can take the benefits of both solid polymer electrolytes and liquid electrolytes, exhibiting outstanding room‐temperature ionic conductivity higher than 10^−4^ S cm^−1^.^[^ ^30^, ^31^, ^32^, ^33^ ^]^ For example, Li et al. designed a dual‐salt (lithium bis(trifluoromethanesulfonyl)imide‐lithium hexafluorophosphate, LiTFSI‐LiPF_6_) GPE using carbonic acid organic solvents as plasticizer and obtained a room temperature ionic conductivity up to 0.56 mS cm^−1^.^[^ ^34^ ^]^ Unfortunately, the introduction of volatile organic electrolyte in GPEs would inevitably cause safety risks in terms of overheating and fire. Therefore, developing ionogel electrolytes based on non‐flammable ionic liquid (IL) instead of volatile liquid in GPEs has been regarded as a reassuring routine to diminish safety concerns.^[^ ^35^ ^]^ However, addressing these safety risks of GPEs without sacrificing desirable ionic conductivity still remains a great challenge. In ionogel electrolytes, the Li^+^ ions would coordinate with anions from lithium salt to form negatively charged clusters. Then, Li^+^ ions would migrate together with their first coordination shell, known as slow vehicular transport mechanism, thus resulting in poor cycle capability and serious polarization of lithium batteries.^[^ ^36^ ^]^

Introducing zwitterions into ionogel electrolytes could effectively optimize the local Li^+^ coordination environment to improve Li^+^ transport kinetics and bulk/interfacial structural stability.^[^ ^37^, ^38^ ^]^ Generally, the zwitterion could boost Li salt dissociation and meanwhile provide additional Li^+^ coordination sites, resulting in more mobile Li^+^. However, zwitterion and TFSI^−^ differ in the ability to bond with lithium ions due to their different electronegativity, leading to Li^+^ ions preferentially binding to species with stronger nucleophilicity. The real binding sites of Li^+^ ions and the corresponding Li^+^ conduction mechanism in zwitterion‐based ionogel cannot be clarified. What's worse, the coordination model between Li^+^ and TFSI^−^ has been reported to present several variants under different lithium salt concentration, including free TFSI ions, contact ion pairs (TFSI ions interacting with a single Li ion), and aggregated ion pairs (TFSI ions surrounded by two or more Li ions, similar with quasi crystal).^[^ ^39^, ^40^ ^]^ The related Li^+^ binding energies of TFSI^−^ are different in the three coordination models, further complicating the tunability on Li^+^ coordination environment. Therefore, it is of urgency and challenge to develop an efficient strategy to investigate the interaction between Li^+^ and TFSI^−^/zwitterion and clarify ion transport mechanism of ionogel electrolytes.

Herein, we fabricate a zwitterion‐based copolymer matrix based on the copolymerization of trimethylolpropane ethoxylate triacrylate (ETPTA) and 2‐methacryloyloxyethylphosphorylcholine (MPC, the zwitterion selected in this work). ETPTA with trivalent vinyl groups is promising for generating cross‐linked structure, contributing to strengthened thermal and mechanical strength.^[^ ^33^, ^41^ ^]^ The MPC with phosphonate anions displays proper ion‐pairing of Li‐ion, thus providing effective regulation on Li^+^ coordination architecture compared with carboxylate and sulfonate anions.^[^ ^37^, ^42^, ^43^ ^]^ The polymer phase is fixed with LiTFSI‐contained IL1‐butyl‐3‐methylimidazolium bis(trifluoromethylsulfonyl)imide ([C4mim] [NTf2]) to form homogeneous ionogel. It is worth mentioning that the [C4mim] [NTf2] shows excellent lithium salt solubility, up to 4 m. The interactions between Li^+^ and TFSI^−^/MPC under different salt concentration are investigated, the corresponding Li^+^ binding energies are calculated via density functional theory (DFT). It is shown that the TFSI^−^ and MPC exhibit almost equal binding energies with Li^+^ in their specific coordination architecture, leading to Li^+^ coordination shell jointly occupied by MPC and TFSI^−^. Benefiting from the competitive Li^+^ attraction of TFSI^−^ and MPC, the energy barrier of Li^+^ desolvation is sharply decreased and thus the room‐temperature ionic conductivity can reach a value of 4.4 × 10^−4^ S cm^−1^. Besides, the coulombic interaction between TFSI^−^ and MPC can greatly decrease the reduction stability of TFSI^−^, boosting the in situ derivation of LiF‐enriched solid electrolyte interface (SEI) layer on lithium metal surface. As expected, the assembled Li||LiFePO_4_ cells deliver a high reversible discharge capacity of 139.1 mAh g^−1^ at 0.5 C and good cycling stability (96% capacity retention after 200 cycles at 0.5 C). Besides, the pouch cells exhibit a steady open‐circuit voltage and could operate normally under abuse testing (fold, cut), verifying its outstanding safety performance.

## 2 Results and Discussion

**Figure 1:**
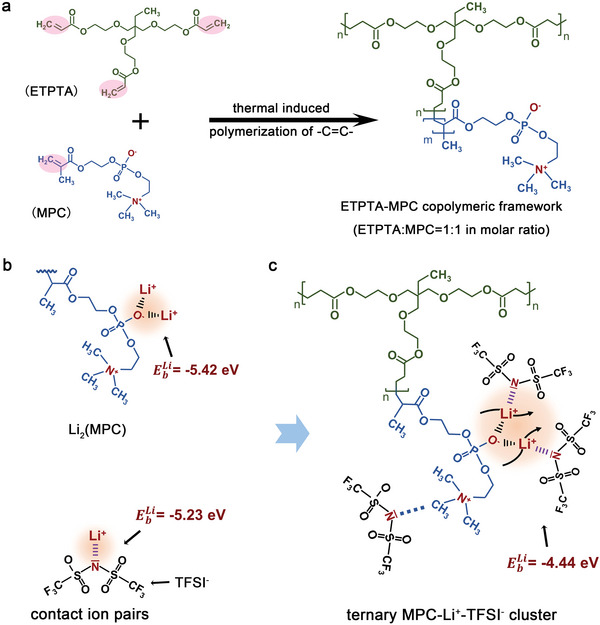
a) The schematic diagram of the polar copolymer preparation process. The polar copolymer framework was prepared by thermal‐induced free radical (—C=C—) copolymerization of trimethylolpropane ethoxylate triacrylate (ETPTA) and 2‐methacryloyloxyethyl phosphorylcholine (MPC). The ionic liquid containing lithium bis(trifluoromethylsulfonyl)imide (LiTFSI) was chosen as plasticizer. b) The schematic illustration of the ion interaction between Li^+^ ions and MPC/TFSI^−^. The almost equal Li^+^ binding energies ($E_{b}^{Li}$) of Li_2_(MPC) and contact ion pairs make TFSI^−^ and MPC jointly occupy the first coordination shell of Li^+^ and form ternary MPC‐Li^+^‐TFSI^−^ cluster. c) The schematic diagram of ternary cluster. The competitive Li^+^ attraction of MPC and TFSI^−^ leads to decreased Li^+^ desolvation barrier and therefore, accelerates the Li^+^ conduction.

**Figure** **1** shows the fabrication process of the polar polymeric framework (ETPTA‐MPC copolymer) and the local coordination model between Li^+^ ion and MPC/TFSI^−^. First, the ionogel electrolyte featuring polar polymeric framework was prepared by mixing thermal curing agent ETPTA and MPC (1:1 molar ratio) into LiTFSI‐contained IL, followed by thermal curing process. With 2,2'‐Azobis(2‐methylpropionitrile) as the initiator, the in situ curing process of liquid precursor occurs based on the thermal‐induced polymerization of terminal alkene units (—C=C—) of MPC and ETPTA (Figure 1a). The polymerization of ETPTA and MPC was evidenced by Fourier‐transform infrared spectroscopy (FTIR), as shown in Figure S1 (Supporting Information), the characteristic peaks of the —C=C— bond at ≈1610–1640 cm^−1^ disappears after the thermal curing process at 80 °C for 4 h.^[^ ^44^ ^]^ The X‐ray diffraction pattern of copolymer exhibits two broad peaks at 12° and 19°, which are distinguished with the diffraction results of poly(ETPTA) and poly(MPC) (Figure S2a, Supporting Information). The newly formed polymer phase may result from the copolymerization of MPC and ETPTA. Besides, the^1^H Nuclear magnetic resonance spectrometer (NMR) spectrums of poly(ETPTA), poly(MPC), and poly(ETPTA‐MPC) are shown in Figure S2b (Supporting Information). In the^1^H NMR result of poly(ETPTA‐MPC), the main peak of poly(ETPTA) at 3.34 ppm (in pink circle) disappears and the splitting peak at 3.82 ppm (in green circles) differs from that of poly(MPC). These results demonstrate the formation of a copolymer phase, rather than a two‐phase mixture. It is worth noting that the outstanding ability of ETPTA monomer to self‐associate and form physical cross‐links endows the copolymer framework with superior IL‐keeping ability (≈80% volume fraction of IL in the precursor), which is vital for obtaining electrolytes with desirable ionic conductivity and proper elasticity. The mechanical properties of ionogels were evaluated through nanoindentation tests. As shown in Figure S3 and Table S2 (Supporting Information), the Young's modulus and Hardness decrease with the relative content of IL. The decreased rigidity of ionogel is superior in relieving the interface stress caused by electrode volume expansion during charge/discharge cycles. Meanwhile, the inserted MPC molecular functionalized the polymer chain via two aspects: 1) the negatively charged phosphate group ($-PO_{4}^{-}$) of MPC would serve as Li^+^ acceptor and is involved in Li^+^ conduction. According to the number of Li^+^ ions that coordinate with MPC, the coordination models between Li^+^ and MPC are divided into two types: Li(MPC) (each MPC coordinates with one Li^+^) and Li_2_(MPC) (each MPC surrounded by two Li^+^ ions after further lithiation); 2) The cationic quaternary amines (R_4_N^+^) could restrict the mobility of TFSI^−^ anions by coulombic interaction, contributing to higher ion transference number ($t_{Li^{+}}$).

As shown in Figure 1b, both TFSI^−^ anions and MPC in this system serve as acceptor for Li^+^ ions via coulombic interaction. When MPC coordinates with Li^+^ ion in form of Li_2_(MPC) after a deep lithiation, it shows Li^+^ binding energy ($E_{b}^{Li}$) of −5.42 eV by DFT calculation. What's more, the contact ion pairs of Li^+^ and TFSI^−^ exhibit similar $E_{b}^{Li}$ value of −5.23 eV, proving the similar ability of MPC and TFSI^−^ to bond with lithium ions. Therefore, MPC and TFSI^−^ would equally occupy the first coordination shell of Li^+^ ions in forms of Li_2_(MPC) and contact ion pairs, respectively. Thus, ternary MPC‐Li^+^‐TFSI^−^ clusters are formed along the zwitterion‐based copolymeric framework. To verify the benefits of the competitive Li^+^ coordination between MPC and TFSI in enhancing Li^+^ transport kinetics, the $E_{b}^{Li}$ of the ternary cluster is also calculated, which is decreased to −4.44 eV. The decreased $E_{b}^{Li}$ value indicates that the competitive Li^+^ attraction behavior between MPC and TFSI^−^ can significantly decrease the desolvation barrier of Li^+^ ions. The weaker interaction with anions enables the trapped Li^+^ escape to be mobile ions more easily.

**Figure 2:**
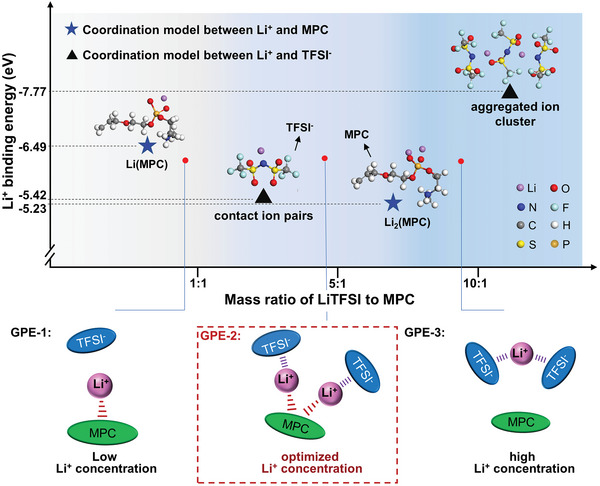
Coordination models between Li^+^ and TFSI^−^/MPC under different mass ratio of LiTFSI and MPC, and the corresponding binding energies obtained by DFT calculations. The coordination models between Li^+^ and MPC are divided into two types: Li(MPC) (MPC coordinated with one Li^+^ ion) and Li_2_(MPC) (MPC is surrounded by two Li^+^ ions). Similarly, the coordination models between Li^+^ and TFSI^−^ also present as two types: contact ion pairs (TFSI^−^ binds with one Li^+^ ion) and aggregated ion clusters (TFSI^−^ binds with two or more Li^+^ ions). For the binding energies of Li^+^‐MPC and Li^+^‐TFSI^−^ pairs, three samples with different Li^+^ coordination environment are denoted as GPE‐1, 2, 3. In GPE‐1, as the $E_{b}^{Li}$ value of Li(MPC) is greatly higher than that of contact ion pair, the limited Li^+^ ions would preferentially bind with MPC in forms of Li(MPC) while leaving TFSI^−^ as free anions. In GPE‐2, MPC and TFSI^−^ jointly occupy the first coordination shell of Li^+^ as Li_2_(MPC) and the contact ion cluster exhibit similar equal $E_{b}^{Li}$. In GPE‐3, the $E_{b}^{Li}$ values of Li_2_(MPC) and the aggregated ion clusters are calculated to be −5.23 and −7.77 eV, indicating that TFSI^−^ possess stronger coulombic interaction with Li^+^ than MPC in concentrated ionogel.

To further explain the formation of the competitive Li^+^ coordination architecture between MPC and TFSI^−^, we demonstrate the general coordination models of Li^+^‐MPC and Li^+^‐TFSI^−^ ion pairs with varing mass ratios of LiTFSI to MPC. These $E_{b}^{Li}$ values were obtained using the $E_{b}$ DFT method. As shown in **Figure** **2**, under low LiTFSI: MPC ratio, the MPC and TFSI^−^ coordinate with Li^+^ ion in forms of Li(MPC) and contact ion pairs, respectively. As the $E_{b}^{Li}$ value of Li(MPC) (−6.49 eV) is greatly higher than that of contact ion pair (−5.42 eV), the limited Li^+^ ions would preferentially bind with MPC in form of Li(MPC) while leaving TFSI^−^ as free anions (denoted as GPE‐1). Under high LiTFSI: MPC ratio, MPC would be further lithiated to present as Li_2_(MPC), and TFSI^−^ anions would form aggregated ion clusters with concentrated Li^+^ ions. The corresponding $E_{b}^{Li}$ values of Li_2_(MPC) and the aggregated ion clusters are calculated to be −5.23 eV and −7.77 eV, indicating that TFSI^−^ possess stronger coulombic interaction with Li^+^ than MPC in ionogel. Therefore, in GPE‐3, most Li^+^ ions are snatched by TFSI^−^ to form aggregated ion clusters. The formation of aggregated ion cluster accounts for the increased $E_{b}^{Li}$ value of Li^+^‐TFSI^−^ system with salt concentration increasing. Meanwhile, the decreased $E_{b}^{Li}$ of Li^+^‐MPC pair is thought a result of reduced local electron density after further lithiation of MPC. The desirable ionogel in which MPC and TFSI^−^ jointly occupy the first coordination shell of Li^+^ could only be fabricated at proper LiTFSI: MPC ratio as Li_2_(MPC) and contact ion pairs exhibit similar $E_{b}^{Li}$ values. Furthermore, the ion‐ion interaction was deeply discussed by multispectral characterization to provide experimental evidence for the above theoretical prediction.

**Figure 3:**
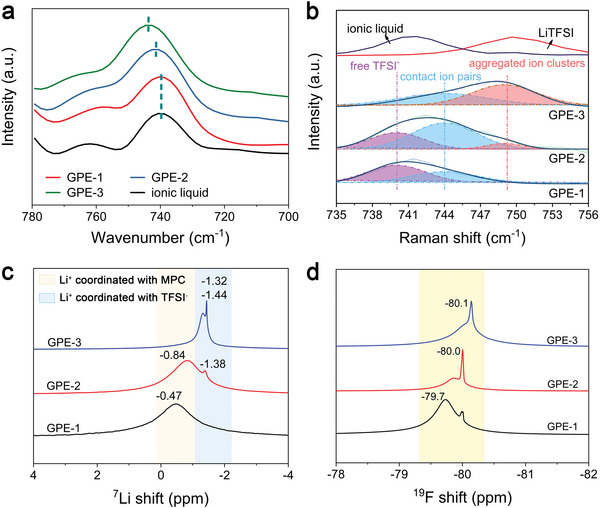
The multispectral characterization for clarifying the fabrication of ternary cluster. S–N–S vibration peak in a) FTIR and b) Raman of ex‐formed GPE‐1, 2, and 3 in which TFSI^−^ species exist as free anions, contact ion pairs and aggregated ion cluster respectively. The purple, blue, and pink fitting peaks in Raman result represent free TFSI^−^, contact ion pairs and aggregated ion cluster respectively. c) ^7^Li and d) ^19^F NMR spectra corresponding to the above samples. As expected, the ternary cluster is finally demonstrated to be fabricated in GPE‐2.

In the experiment section, the GPE‐1, 2, and 3 featuring different ion coordination architectures were selected from a series of ionogels with different concentrations of lithium salt (group 1) and the relative content of IL (group 2). The detailed preparation process and the composition of GPE‐1, 2, and 3 are shown in the Supporting Information. The ion coordination between Li^+^ and TFSI^−^ in GPE‐1, 2, and 3 were clarified based on FTIR and Raman spectrum methods by gaining insight into the peak shifting of S–N–S functional group (belonging to TFSI^−^) between 740 and 749 cm^−1^. As shown in **Figure** **3**a, compared with the peak location of IL, in which TFSI exist in free state, the deviation values of S–N–S vibration in GPE‐2 and GPE‐3 is ≈1.9 and 3.8 cm^−1^, respectively. While no obvious peak shifting could be observed in GPE‐1, proving that TFSI^−^ in GPE‐1 mainly exists as free anions. Meanwhile, the increased wavenumbers in GPE‐2 and GPE‐3 suggest the gradual decrease of the bond length of S–N–S. The bond lengths of S–N–S in free TFSI^−^, contact ion pairs and aggregated ion clusters are calculated to be 1.637, 1.624, and 1.612 Å, respectively (Figure S4, Supporting Information), which is consistent with the above FTIR results. Similar to FTIR, the peak location of characteristic functional group in Raman could also provide information for the coordination model between Li^+^ and TFSI^−^. It is previously reported that S–N–S vibration could be divided into three peaks at 740, 744, and 749 cm^−1^, corresponding to free TFSI^−^, contact ion pairs, and aggregated ion clusters, respectively.^[^ ^40^ ^]^ The fitting results of Raman spectrum are shown in Figure 3b, which provide an intuitionistic evidence for the coordination state of TFSI^−^ in the three ionogels. Based on the analysis results of FTIR and Raman spectrum, the TFSI^−^ anions in GPE‐1, 2, and 3 are dominated by free anion, contact ion pairs, and aggregated ion clusters, respectively, which is consistent with the theoretical prediction based on DFT calculation. The detailed FTIR and Raman results of GPEs are displayed in Figures S6 and S7 (Supporting Information).

The corresponding ion‐dipole interaction between Li^+^ and MPC is verified by FTIR and NMR. First, the P–O stretching vibrational band in FTIR spectrum (Figure S8, Supporting Information) shift from 962 to 972 cm^−1^ when LiTFSI is added to the mixture of ETPTA‐MPC‐IL. This result illustrated an attractive interaction between $-PO_{4}^{-}$ and Li^+^. Besides, as for the NMR spectra shown in Figure 3c, the broad peaks in^7^Li spectrum located between −1 and 1 ppm are related to Li^+^ ions that coordinated with MPC,^[^ ^37^ ^]^ and the broad linewidth indicates an anisotropic Li^+^ coordination environment at MPC. The^7^Li spectrum of GPE‐1 exhibits only a broad peak at ‐0.47 ppm, which is thought as the characteristic peak of Li(MPC). As the TFSI^−^ anions in GPE‐1 have been proved in free state by FTIR and Raman spectrum, it is thought that MPC preferentially occupies the first coordination shell of Li^+^ ions under low salt concentration. It is worth noting that there are two peaks at ‐0.84 and ‐1.38 ppm in the NMR results of GPE‐2 and the peak between −2 and −1 ppm corresponds to Li^+^ ions bonded with TFSI^−^ anions.^[^ ^45^ ^]^ This means two distinguished Li^+^ coordination environments in the GPE‐2. The Li peak at −1.38 ppm is related to Li^+^ ions coordinated with TFSI^−^ anions. Besides, compared with the Li peak in GPE‐1, the chemical shift of Li^+^ at −0.84 ppm exhibits an obvious upfield shift, suggesting the increased electron density around the Li^+^ nucleus. It is concluded that the Li^+^ ions coordinating with MPC were affected by adjacent TFSI anions, in other words, TFSI anions occupy the coordination shell of Li^+^ together with MPC.^[^ ^37^ ^]^ The coexistence of characteristic peaks belongs to contact ion pairs and Li_2_(MPC) has demonstrated that TFSI^−^ and MPC equally serves as Li^+^ acceptor with similar binding energies in GPE‐2. In addition, the peaks related to Li^+^‐MPC interaction disappears in the result of GPE‐3 and meanwhile, the characteristic peak at about −1.4 ppm is differentiated into shoulder peaks, corresponding to contact ion pairs (−1.32 ppm) and aggregated ion clusters (−1.44 ppm), respectively. The narrow linewidth of the peak at −1.44 ppm illustrates an isotropous distribution of local Li environment in the aggregated ion clusters. The NMR analysis provides a detailed evidence for the changeable ion interaction in ionogel with different Li salt concentration and agrees well with the above prediction. The^19^F spectra serving as supplementary evidence for reflecting the interaction between Li^+^ and TFSI^−^ are shown in Figure 3e. The upfield shift of the NMR peaks from −79.7 to −80.2 ppm indicates an enhanced Li^+^‐ TFSI^−^ coordination, agree well with the FTIR and Raman analysis.^[^ ^46^ ^]^

**Figure 4:**
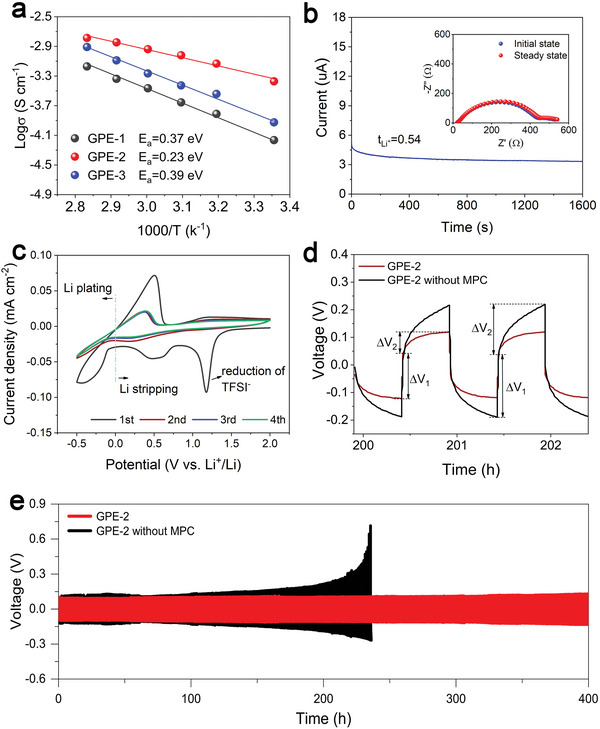
Electrochemistry tests for Li^+^ transport and high‐voltage/cycle stability. a) Arrhenius plots of SS|GPEs|SS cell with different ion configurations. b) Polarization curve and impedance diagram of the cell before and after polarization (the inset) for Li|GPE‐2|Li cell at 25 °C. c) CV curves of Li|GPE‐2|SS battery at a scanning rate of 1 mV s^−1^ at 25 °C. d) Locally enlarged view at selected cycles of e) Voltage profiles of the Li|GPE‐2|Li battery and the corresponding control group (without MPC) at current densities of 0.1 mA cm^−2^.

To evaluate the impact of ion coordination on Li^+^ conduction, the Nyquist plots of GPE‐1, 2, and 3 were measured and shown in Figure S9 (Supporting Information). The room‐temperature ionic conductivity (*σ*) is calculated based and Equation S1 (Supporting Information) and shown in Figure S10 (Supporting Information).^[^ ^47^ ^]^ The exceedingly higher *σ* of GPE‐2 (4.4 × 10^−4^ S cm^−1^) at room temperature surpasses those of GPE‐1 (0.82×10^−4^ S cm^−1^) and GPE‐3 (1.03×10^−4^ S cm^−1^). Besides, the activation energy (*E*_a_) of Li^+^ transport is obtained by calculating *σ* as a function of temperature based on the Arrhenius formula (Equation S2, Supporting Information). As shown in **Figure** **4**a, the GPE‐2 exhibit the lowest *E*_a_ value of 0.23 eV. The decreased *E*_a_ may benefit from the competitive Li^+^ attraction between MPC and TFSI^−^. In addition, the Arrhenius linearity behavior of ionic conductivity was observed when the temperature rose from 25 to 80 °C. This indicates that the Li^+^ ions could desolvate from coordination shells and migrate through re‐coordination along the polymer backbone.^[^ ^48^ ^]^ Unfortunately, except for the contact ion pairs in the ternary cluster, there exist free‐state contact ion pairs in the space between the polar polymeric segment. In order to exclude the contribution of free‐state contact ion pairs in GPE‐2 to the outstanding ionic conductivity, we set a control group in which MPC was not added. The NMR results demonstrate the predomination of contact ion pairs in the control group (Figure S11, Supporting Information). As shown in Figure S12 (Supporting Information), the room‐temperature ionic conductivity of the control group was measured to be 2.4 × 10^−4^ S cm^−1^, which is slightly higher than those of GPEs‐1 and GPEs‐3 but still significantly lower than that of GPEs‐2. Therefore, it is the competitive Li^+^ coordination between MPC and TFSI^−^ that contributes most to the enhanced ion transport kinetics. Meanwhile, the measured *E*_a_ value (0.33 eV) of the control group delivers a similar conclusion.

The ion–dipole interaction between TFSI^−^ anions and MPC could result in an improved $t_{Li^{+}}$ in GPEs, which is another crucial factor to evaluate the effective ionic conductivity. Solid‐state electrolytes with high $t_{Li^{+}}$ have been demonstrated effective to suppress the dendrite formation by prolonging the critical nucleation time of lithium dendrite.^[^ ^47^ ^]^ Besides, the high $t_{Li^{+}}$ has been proved to play a positive role in accelerating the complete delithiation of active material in cathode.^[^ ^49^ ^]^ The $t_{Li^{+}}$ of the GPE‐2 electrolyte was obtained by using potentiostatic polarization method, before and after which electrochemical impedance spectroscopy (EIS) measurements was applied to a symmetric cell immediately. As the direct current (DC) polarization results shown in Figure 4b, $t_{Li^{+}}$ of GPE‐2 was calculated to be 0.54 on basis of the Bruce–Vincent–Evans equation (Equation S3, Supporting Information), which is higher than that of IL (≈0.2) and most PEO‐based solid electrolyte (<0.4). The high $t_{Li^{+}}$ of GPE‐2 could be interpreted as the result of that, besides the improved Li^+^ migration, the movement of TFSI^−^ is further restricted by the cationic quaternary amines (R_4_N^+^) of MPC by coulombic attraction. The interaction between R_4_N^+^ and TFSI anions is proved by analyzing the peak shift of C‐N bond. As shown in Figure S13 (Supporting Information), the peak related to C—N bond shift from 1177 to 1181 cm^−1^ after mixing MPC with IL, indicating an interaction between R_4_N^+^ group and TFSI in IL. Besides, as shown in Figure S14 (Supporting Information), the $t_{Li^{+}}$ of GPE‐1 and GPE‐3 are calculated to be 0.39 and 0.45 respectively, which may be a result of lower Li^+^ conductivity compared with GPE‐2. As for the improvement $t_{Li^{+}}$ of GPE‐3 to GPE‐1, it is concluded that the movement of TFSI^−^ in GPE‐3 is further restricted due to the formation of aggregate ion clusters. In addition, the linear sweep voltammetry (LSV) measurement was carried out to prove the excellent high‐voltage stability (Figure S15, Supporting Information). The result shows no apparent current fluctuation ahead of 5.1 V, which could be regarded as the credit of good electrochemistry stability of polymer components.

Figure 4c shows the reversible Li plating and stripping process in Li|GPE‐2|Cu. Besides, the reduction and oxidation behaviors of ionogel electrolytes were also reflected by cyclic voltammetry (CV) curves. The prominent reduction peak between 1.2 and 1.4 V corresponds to the reduction of TFSI^−^, which is promising for generating LiF‐enriched solid electrolyte interface (SEI) layer on Li anode surface, thus leading to stable electrode/electrolyte interface. However, the reduction peak of TFSI^−^ could hardly be observed in the CV curve of the control group (Figure S16, Supporting Information), demonstrating the positive effect of the ion‐ion interaction between TFSI^−^ and MPC on accelerating TFSI^−^ decomposition.^[^ ^50^ ^]^ Further, the voltage profiles pertaining to Li plating/stripping for 0.5 h at a current density of 0.1 mA cm^−2^ were measured in the symmetric Li|GPE‐2|Li cell to verify the superiority of MPC addition on enhancing interface stability of Li metal batteries (Figure 4e). The symmetrical cell presents an initial polarization of 91 mV, and could remain stable without obvious fluctuation for more than 400 h. While a voltage surge occurs at around 230 h in the voltage profile of the control group in which MPC was absent from the system. The enhanced reversibility of Li|GPE‐2|Li batteries should be benefited from the fast Li^+^ transport kinetics of the electrolyte and the stable SEI layer. The enlarged voltage profiles for GPE‐2 and the control group are shown in Figure 4d. As reported, the total overpotential could be divided into two parts. The ohmic drop (△*V*_1_) is related to the initial resistance while △*V*_2_ is thought to reflect the ionic concentration polarization.^[^ ^36^ ^]^ It is observed that the△*V*_2_ value of Li|GPE‐2|Li is smaller than that of the control group, indicating the enhanced Li^+^ mobility and higher $t_{Li^{+}}$ arising from TFSI^−^‐MPC interaction. The cycle performance of Li|GPE‐1|Li and Li|GPE‐3|Li cells were also tested and the voltage profiles are shown in Figure S17 (Supporting Information). Due to the accelerated TFSI^−^ decomposition kinetics, both GPE‐1 and 3 show improved interfacial stability with Li anode compared with the control group. But the cycle performance of Li|GPE‐1|Li and Li|GPE‐3|Li are still poorer than Li|GPE‐2|Li cells. Apart from the ionic conductivity, differences in Li^+^ concentration, viscosity, and elasticity properties may influence the interfacial stability.

**Figure 5:**
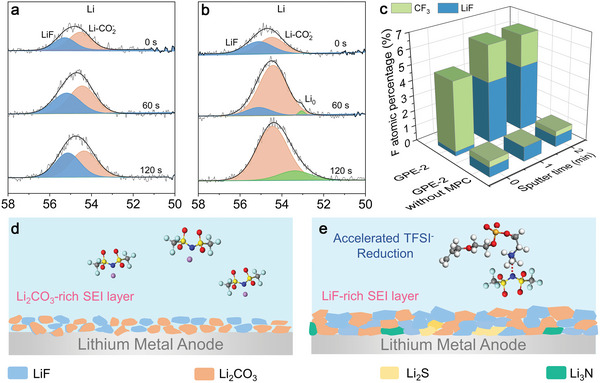
Analysis of the in situ formed SEI layer. Li spectra of the SEI layer in Li–Li symmetric battery assemble with a) GPE‐2 and b) control group after different XPS sputter etching time (0, 60, and 120 s from top to bottom). c) A depth profiling of F atomic percentage in SEI layer and the ratio of different F species (i.e., CF_3_ and LiF). d,e) The schematic diagram of specific ionic coordination on accelerating dense SEIs formation.

The components and distribution of SEI layer were investigated by applying X‐ray photoelectron spectroscopy (XPS) methods on the cycled Li metal surface. With the assistance of Ar beam etch, the XPS depth profiles of SEI layer were obtained. As shown in **Figure** **5**a,b, Li spectra were selected to identify the composition of SEI layer. The fitting results shown in Figure 5a indicates that LiF predominate in the SEI layer in Li|GPE‐2|Li cell. Besides, in the F1s spectra (Figure S18, Supporting Information), the peak of LiF (684.8 eV) appears in the surface of SEI and its content gradually increases up to the surface of Li anode. The same phenomenon is observed in N and S spectra, in which Li_3_N and Li_2_S phase are only detected in the inner SEI layer. Furthermore, Figure 5c displays the percentage of F species at different etching depth. As shown, the percentages of LiF are 62.8% and 67.3% of F element at 10 and 20 nm depth of SEI in Li|GPE‐2|Li cells, while it is only 5.8% in surface layer. The obtained XPS results demonstrate that the anion‐derived inorganic SEI layer could effectively suppress interfacial side‐reaction. However, in the control group, it is observed that the LiF content at different etching depth is negligible (Figure 5b). The Li_2_CO_3_ phase, which predominates in inner SEI layer of the control group, may be the result of electrode oxidation in the characterization process. Similarly, as shown in Figure S19 (Supporting Information), there is no characteristic peaks of Li_3_N and Li_2_S detected on the SEI layer of the Li|GPE‐2 without MPC|Li cells. What is more, the presence of Li^0^ at superficial depth (10 nm) indicates the crumbly SEI layer in the control group, leading to a failure in protecting lithium metal anode. Based on the analysis of SEI components and CV curve, the introduction of MPC decreases the reduction stability of TFSI^−^ and give a significant impact on its decomposition behavior (Figure 5d,e).

**Figure 6:**
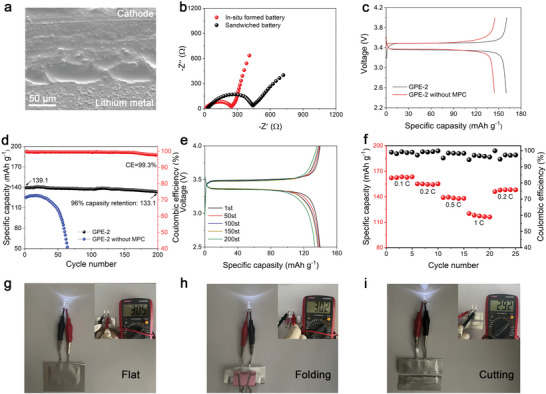
Evaluation of practical performance of the assembled full cells. a) SEM image in the cross section of in‐situ assembled Li|GPEs|LFP cell. b) Electrochemical impedance plots of the devices. c) The charge and discharge curve at 0.1 C and d) the long‐time cycling performance of Li|GPE‐2|LFP cells at 0.5 C. e) The charge–discharge curve from 1 to 200 cycles, indicates a slight fading of capacity. f) The rate performances of the cell. g–i) The open‐circuit voltages of the pouch cell under different states and the optical images of the pouch cell lighting a green LED under different states.

To evaluate the lithium storage performance of Li|GPE|LiFePO_4_ batteries, the coin cells assembled with GPE‐2 electrolytes were thermostatically recharged and discharged in a voltage range of 2.5–4.0 V at 40 °C. Prior to the electrochemistry tests, the cross‐section scanning electron microscopy (SEM) image of the coin cell shown in **Figure** **6**a directly demonstrates the improvement of interfacial contact benefiting from in‐situ polymerization method. Only ambiguous electrode/electrolyte interface could be observed due to the thorough infiltration of flowing liquid precursor into cathode before thermal curing. The in‐situ assembled battery exhibit decreased interface impedance compared with sandwiched battery, reflecting the advantage of in‐situ formed electrode/electrolyte interface in improving interfacial Li^+^ transfer (Figure 6b). The galvanostatic charge–discharge tests were carried out. Typical charge–discharge voltage profiles of the cells at 0.1 C are presented in Figure 6c. The Li|GPE‐2|LiFePO_4_ cells could deliver high discharge capacity of 160.2 mAh g^−1^, 94% theoretical value of 170 mAh g^−1^, while the control group only achieves discharge of 144 mAh g^−1^. Furthermore, the cells assembled with GPE‐2 electrolyte show a lower Ohmic drop than the control group. These results clearly indicate the benefits of GPE‐2 with higher $\sigma_{Li^{+}}$ and $t_{Li^{+}}$, which facilitates the complete delithiation of the active material and decrease the internal impedance. Meanwhile, the batteries exhibit outstanding cycling performance and Coulombic efficiency as shown in Figure 6d. After 200 cycles at 0.5 C, the capacity retention reaches 96% of the initial discharge capacity of 139.1 mAh g^–1^ with Coulombic efficiency (CE) above 99.3%. As Figure 6e shows, even after 200 recycles, no obvious increase of ohmic polarization could be observed in the charge–discharge curve, while the specific discharge capacity of control group shows a catastrophic plunge after only 60 cycles. In addition, Li|GPE|LiFePO_4_ batteries shows satisfactory rate performance, in which average capacity retentions are remained 73% when the rate is increased by 10 times from 0.1 C to 1 C (Figure 6f). It is believed that the improved Li^+^ transport kinetics and the homogeneous SEI layer are of great importance for promoting cycle performance. In addition, the in situ ionogel electrolytes composed of inflammable IL also endow the devices with excellent safety performance, which cannot be ensured when using liquid electrolytes or traditional PEO‐based polymer electrolytes. As illustrated in Figure 6g, the assembled pouch cells can light up a light emetting diode (LED) even after severe folding and cutting tests. Besides, the open‐circuit voltage has no significant decrease. Besides, a video demonstrating the excellent fire‐retardant property of ionogel electrolytes is provided in Supporting Information (Video S1, Supporting Information).

## 3 Conclusions

Based on the multispectral characterization and DFT calculation, we have demonstrated that the Li^+^ binding energy of MPC additive and TFSI^−^ in ionogel electrolyte could be adjusted to similar level by optimizing salt concentration. Thus, MPC and TFSI^−^ could equally occupy the first coordination shell of Li^+^ ion to form ternary MPC‐Li^+^‐TFSI^−^ clusters. Benefiting from the competitive Li^+^ attraction behavior of TFSI^−^ and MPC, the Li^+^ desolvation barrier is sharply decreased. Therefore, the fabricated ionogel exhibits a desirable room‐temperature ionic conductivity of 4.4×10^−4^ S cm^−1^. Additionally, the coulombic interaction between TFSI^−^ and the cationic quaternary amines (R_4_N^+^) of MPC may decrease the reduction stability of TFSI^−^, accelerating the generation of LiF‐enriched SEI layer on lithium metal surface. As a result, both interfacial Li^+^ transport and side‐reaction suppression are improved, contributing to outstanding long‐term cycle stability in Li plating/stripping tests (>400 h at current density of 0.1 mA cm^−2^). Finally, the assembled Li|GPE|LiFePO_4_ battery shows remarkable charge/discharge reversibility (CE = 99.3%), stable rate capability, and high capacity retention (96% of initial discharge capacity) after 200 cycles at 0.5 C. We believe that the “competitive Li^+^ coordination” strategy is effective to develop high‐performance ionogel electrolyte for practical lithium metal batteries.

## Conflict of Interest

The authors declare no conflict of interest.

## Supporting information

Supporting Information

Supplemental Video 1

## Data Availability

The data that support the findings of this study are available from the corresponding author upon reasonable request.
