# Supplementary material for: Competitive Li+ Coordination in Ionogel Electrolytes for Enhanced Li‐Ion Transport Kinetics
Source: Adv Sci (Weinh). 2023 Jun 6;10(23):2300226. doi: 10.1002/advs.202300226 (PMC10427361; doi:10.1002/advs.202300226)
Supplement: Supplementary file 1 — Supporting Information [file ADVS-10-2300226-s002.pdf]

ADVANCED SCIENCE
Open Access

Supporting Information

for *Adv. Sci.*, DOI 10.1002/advs.202300226

Competitive Li^+ Coordination in Ionogel Electrolytes for Enhanced Li-Ion Transport Kinetics

*Jiafeng Li, Tao Zhang, Xiaobin Hui, Ruixiao Zhu, Qiqi Sun, Xiaoxuan Li and Longwei Yin**

Competitive Li^+ Coordination in Ionogel Electrolytes for Enhanced Li-ion Transport Kinetics

Experimental Section

1. Material

Ethoxylated trimethylolpropane triacrylate (ETPTA) was purchased from Macklin. 1-Butyl-3-methylimidazolium bis(trifluoromethylsulfonyl)imide ([C4mim] [NTf2]), 2-methacryloyloxyethylphosphorylcholine (MPC) and 2,2'-Azobis(2-methylpropionitrile) (AIBN) initiator were purchased from Sigma Aldrich. A dehydration procedure towards ETPTA and [C4mim] [NTf2] was required before using. AIBN needs to be purified by recrystallization process to obtain needle-shaped white powder. All the reagents, except MPC needs a -20 ℃ low temperature storage in refrigerator, are stored in Ar-filled glovebox with O_2 and H_2O content below 0.5 ppm

2. Synthesis of GPE precursor

The thermal curing agent was prepared by mixing 200 μl ETPTA with 140 mg MPC powder (molar ratio of 1:1) uniformly. Then, 1.2 ml ionic liquid containing LiTFSI was added to the thermal curing agent. Firstly, LiTFSI concentration is changed from 1 to 4 M (**group 1**) to adjust the ion coordination architectures. For another group, the content of ionic liquid is decreased from 1.2 to 0.2 ml (1.2, 0.8, 0.4 and 0.2 ml) while keeping the LiTFSI concentration at 2 M (**group 2**). From the series of samples, ionogels featuring free TFSI, contact ion pairs and aggregated ion clusters are selected and named as GPE-1, 2 and 3 respectively.

The LiTFSI concentration is based on the volume of ionic liquid. By comparing the FTIR and Raman results, the ionogels featuring free TFSI, contact ion pair and aggregated ion clusters are selected and the composition is shown in **Table S1**.

Table S1. Composition of GPE-1, 2 and 3

ETPTA	MPC	Ionic liquid	LiTFSI	state of $TFSI^-$
200 μl	140 mg	0.2 ml	2 M (688 mg)	Free TFSI (GPE-1)
200 μl	140 mg	1.2 ml	2 M (459 mg)	Contact ion pairs (GPE-1)
200 μl	140 mg	1.2 ml	4 M (230 mg)	Aggregated ion clusters (GPE-1)

2. Cell Preparation

For in-situ formed batteries, 0.01g AIBN initiator was added into 1 g gel precursor and mixed them uniformly. Then, 100 μL gel electrolyte precursor was dropped on the cellulose film between the electrodes within a cell. The hydraulic battery packer is used to assemble coin cells (CR 2025), followed by thermostatically heating at 60 ℃ for more than 2h to obtain in-situ solid battery.

The ionogel electrolyte used in sandwiched batteries is disassembled from the in-situ formed SS||SS batteries to exclude the influence of electrolyte thickness.

As for the ex-formed independent membrane prepared for microstructure analysis, the gel precursor was dripped on release liner and heated at the same condition.

For the Li| |LFP cell, a slurry of LFP, super-P, and poly(vinylidene fluoride) (mass ratio of 8:1:1) dissolved in N-methly-2-pyrrolidone was cast onto an aluminum foil. The cast film was dried in a vacuum oven at 80 °C for 24 h. The active material weight of cathode applied in this experiment is ≈3.5 mg cm^{-2}.

3. Materials characterization

The crystal phases of the synthesized polymer were identified by X-ray diffraction method (XRD, Rigaku D/Max-KA) equipped with a Cu Kα source (λ=1.5406 Å) at a step size of 0.02° with 0.5 s dwelling time. The morphologies, structure and elemental distributions of samples were investigated by field emission scanning electron microscopy (FESEM, SU-70) equipped with energy dispersive spectrometry (EDS) at an acceleration voltage of 10 kV. Chemical compositions of the solid electrolyte interface (SEI) on lithium metal surface were further analyzed by high-resolution X-ray photoelectron spectroscopy (XPS) recorded by Thermo SCIENTIFIC ESCALAB Xi+ instrument equipped with a 1486.68 eV Al Kα probe beam. The Ar sputter was carried out with ion energy of 2000 eV. FTIR characterization (Bruker spectrometer TENSOR 27) was used to prove the disappearance of C=C and the variational chemical state of $TFSI^{-}$. Raman spectra were performed on a JY HR800 micro Raman spectrometer with a 633 nm laser as an excitation source to illustrate the ionic interactions. The coordination environment of Li^{+} was investigated using nuclear magnetic resonance (NMR, Bruker AVANCE III HD 600).

4. Electrochemistry tests

The Lithium metal foils were used as both work electrode and counter electrode, with diameter of 10 mm. The batteries for electrochemistry tests were assembled by dripping liquid precursor onto a porous cellulose membrane

between electrolytes in Ar-filled glovebox with O_2 and H_2O content below 0.5 ppm, followed by heating at 80 °C for 2 h to obtain the solid cells. The ionic conductivity (σ) of the GPE was detected by EIS in a frequency range from 1 MHz to 0.1 Hz and a temperature range from 25 to 80 °C with stainless steel (SS) as symmetric electrodes. And the σ was calculated depending on the Equation 1:

$$\sigma = \frac{L}{RS}$$

Where R is the resistant value of ss||ss cells (the intercept on the x-axis in EIS results), L and S are the thickness and area of GPEs. The corresponding was calculated based on EIS results obtained at different temperature and Equation 2 (Arrhenius formula):

$$\sigma(T) = Aexp\left(-\frac{E_a}{RT}\right)$$

where A is the pre-exponential factor, E_a is the activation energy of activated ion-hopping conduction process, and T is the absolute temperature.

The linear sweep voltammetry (LSV) was carried out using the cell configuration of SS||Li cells at the scanning rate of 1 mV s^{-1} and 25 °C. The t_{Li^+} was conducted by direct-current (DC) polarization of the Li||Li symmetric cell with the DC voltage of 10 mV at 25 °C. And t_{Li^+} was calculated by Equation 3:

$$t_{Li^+} = \frac{I_S(\Delta V - I_0 R_0)}{I_0(\Delta V - I_S R_S)}$$

where ΔV is the applied voltage (10 mV), I_0 and I_S are the initial and steady current through the cell, respectively, R_0 and R_S are the initial and steady resistant value of the Li| |Li obtained by AC impendence, respectively.

5. DFT calculation

The first-principles calculations were conducted in Gaussian 09 (G09) program with Becke's three-parameter hybrid method using the Lee-Yang-Parr correlation functional (B3LYP) at 6-311G++G (d, p) level. Frequency analysis was performed to further confirm the ground state of cation-solvent complexes. The binding energy (E_b) between cations and molecule is defined as following:

$$E_b = E_{\mathrm{Complex}} - \mathrm{E}_M - \mathrm{n} \times \mathrm{E}_{\mathrm{molecule}}$$

where $E_{Complexis}$ is the total energy of cation-molecule complex, E_M is the total energy of cation, $E_{molecule}$ is the total energy of solvent, and the n represent the number of molecular in the complex.

Supporting figures

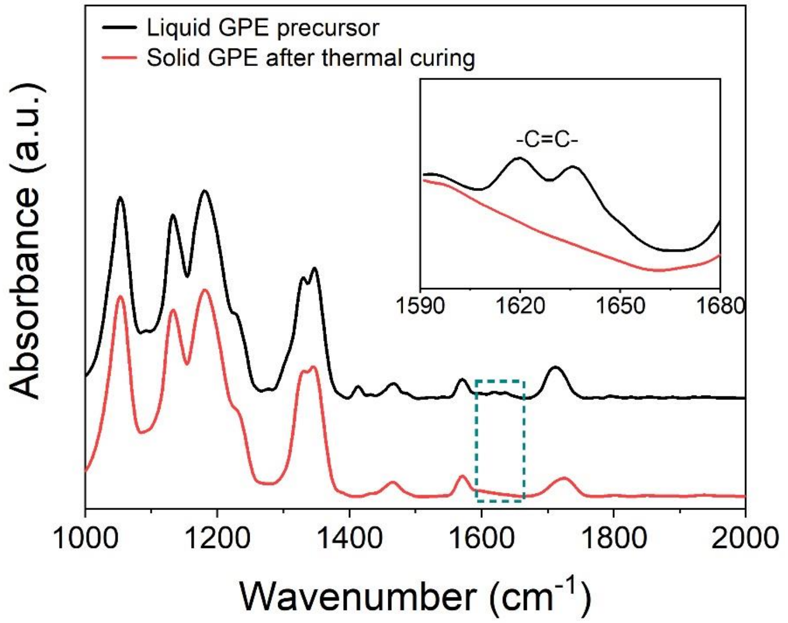

Figure S1. Fourier-transform infrared spectroscopy (FTIR) of liquid precursor and GPE-2 electrolyte.

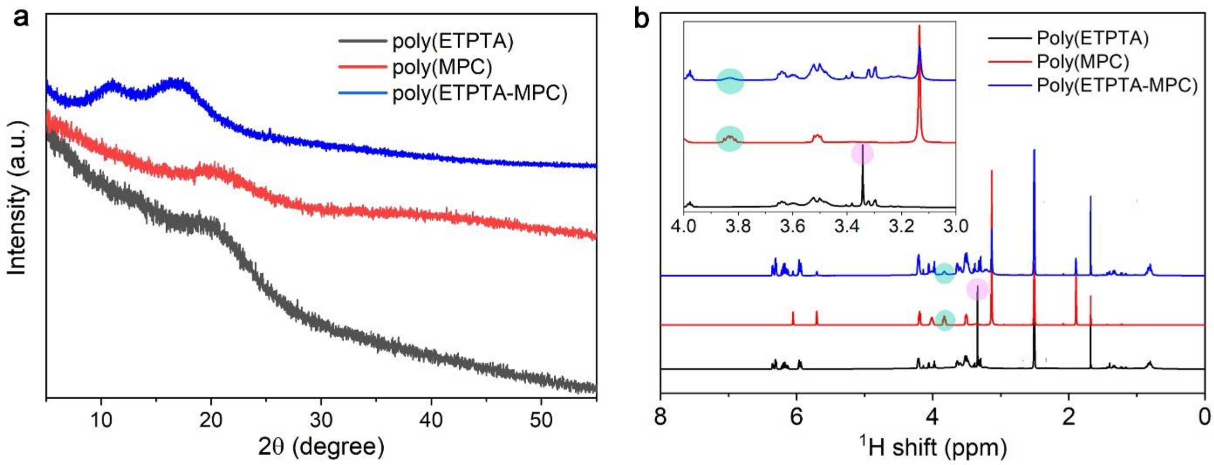

Figure S2. (a) The XRD pattern and (b) ^{1}H NMR spectrums of poly(ETPTA), poly(MPC), and poly(ETPTA-MPC).

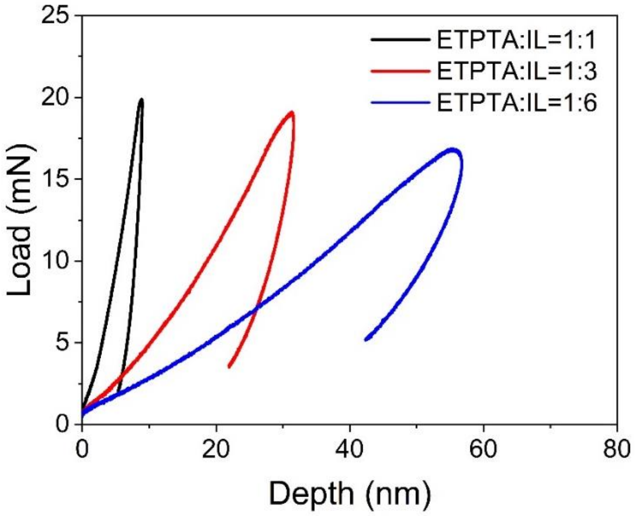

Figure S3. nanoindentation test of ionogels with different ionic liquid percentage (ETPTA:IL=1:1 is based on volume ratio)

Table S2. Reduced Young's modulus and Hardness of ionogels with different ionic liquid contents

Sample number	Volume ratio of ETPTA and MPC	Reduced Young's modulus (MPa)	Hardness (MPa)
1	1:1	210 ± 10	14 ± 0.5
2	1:3	18 ± 2	1.4 ± 0.3

3	1:6	3.6 ± 0.2	0.33 ± 0.03

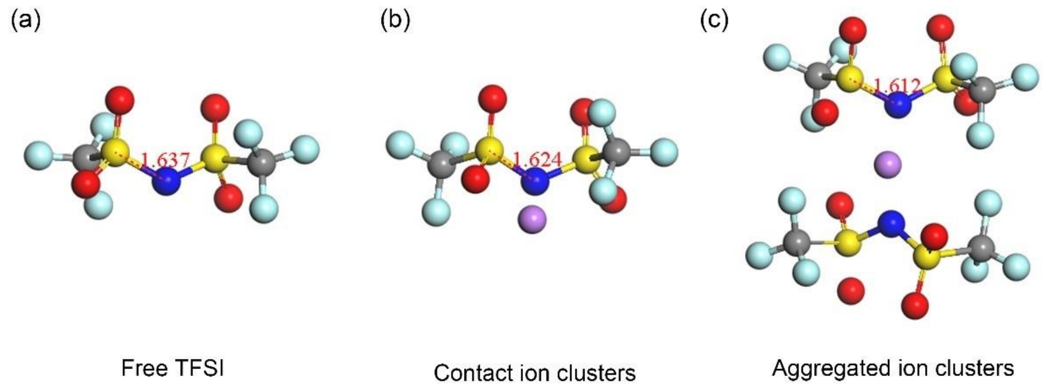

Figure S4. Optimized geometric configurations of free $TFSI^-$, contact ion pairs and aggregated ion clusters.

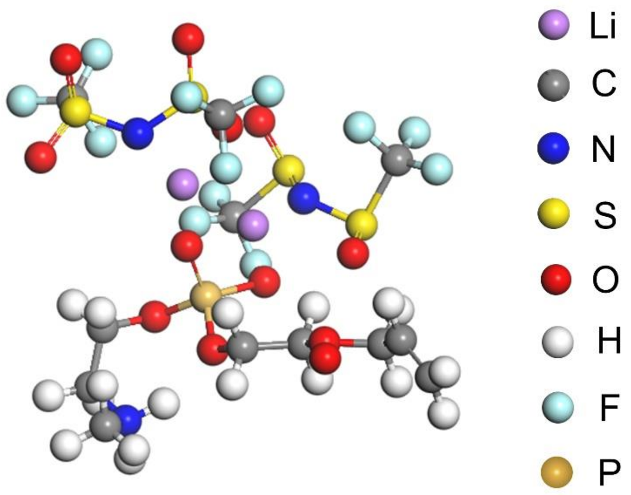

Figure S5. Atomic model of Ternary MPC-Li^+-$TFSI^-$ Clusters.

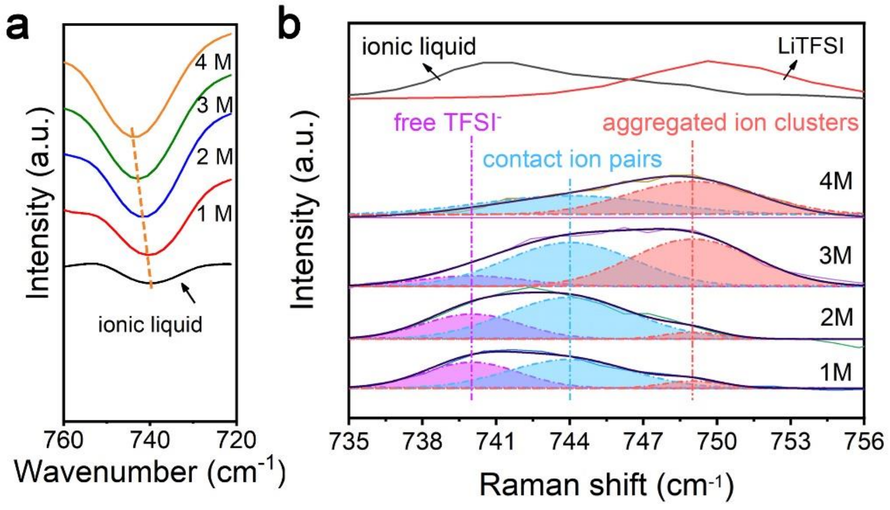

Figure S6. (a) FTIR spectrum and (b) Raman spectra of GPE with different lithium salt concentration (related to **group 1** in **Table S1**), in which the ionic liquid/ETPTA ratio was 6:1. The GPE-2 and GPE-3 were prepared with salt concentration of 2M and 4M respectively.

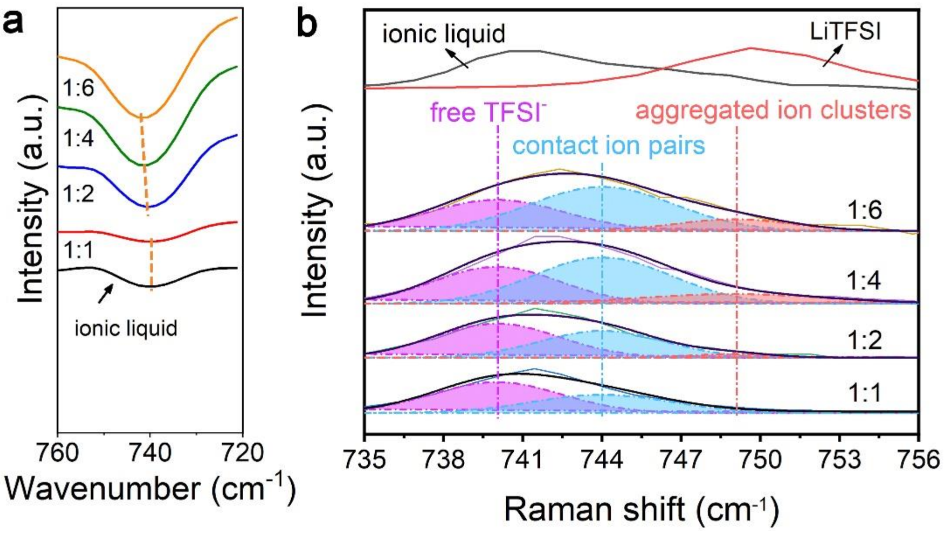

Figure S7. (a) FTIR spectrum and (b) Raman spectra of GPE with different ionic liquid content (related to **group 2** in **Table S2**), in which the salt concentration was 2M. The GPE-1 was obtained when the volume ratio of ionic liquid and ETPTA was decreased to 1:1.

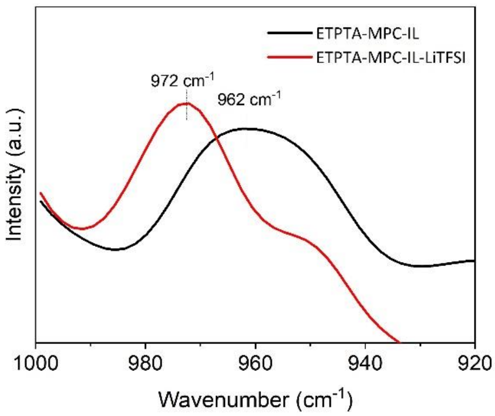

Figure S8. FTIR spectra of ETPTA-MPC-IL with and without LiTFSI

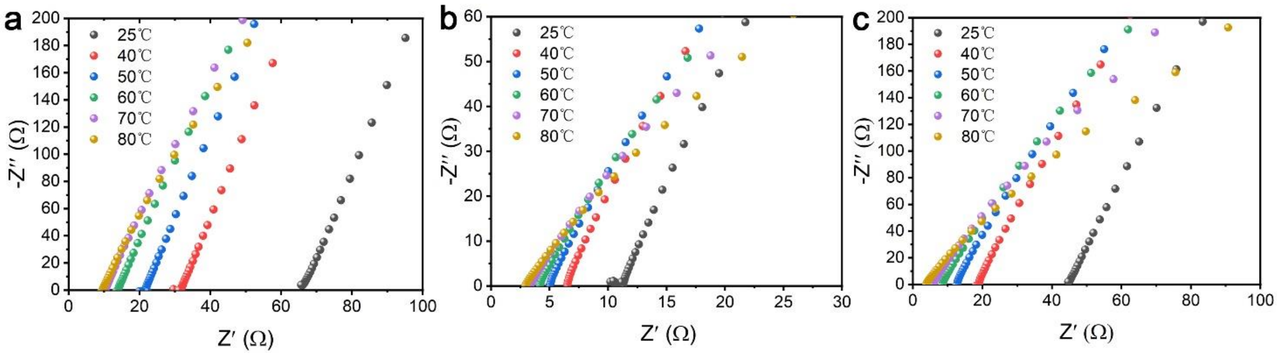

Figure S9. Nyquist plots of the (a) SS|GPE-1|SS, (b) SS|GPE-2|SS and SS|GPE-3|SS cells measured at various temperatures. The diameter of the stainless steel (SS) electrode is 15.6 mm and the thickness of the ionogel electrolyte is about 100 μm.

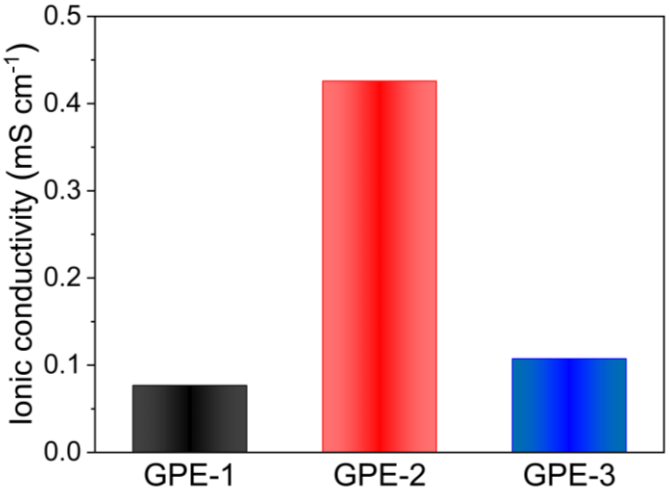

Figure S10. Room-temperature ionic conductivity of GPE-1, 2 and 3.

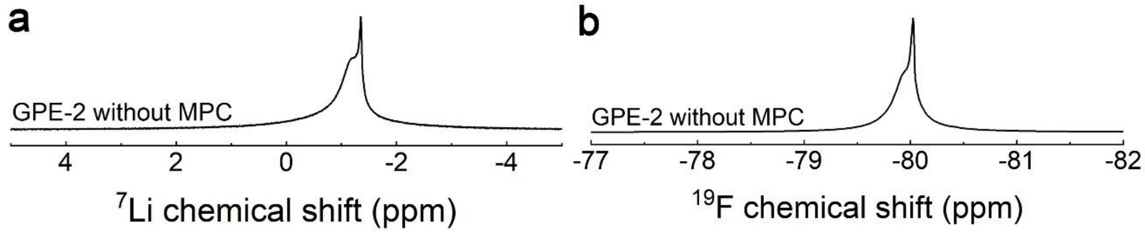

Figure S11. the (a) ^{7}Li and (b) ^{19}F NMR spectra of control group in which MPC was absent (GPE-2 without MPC).

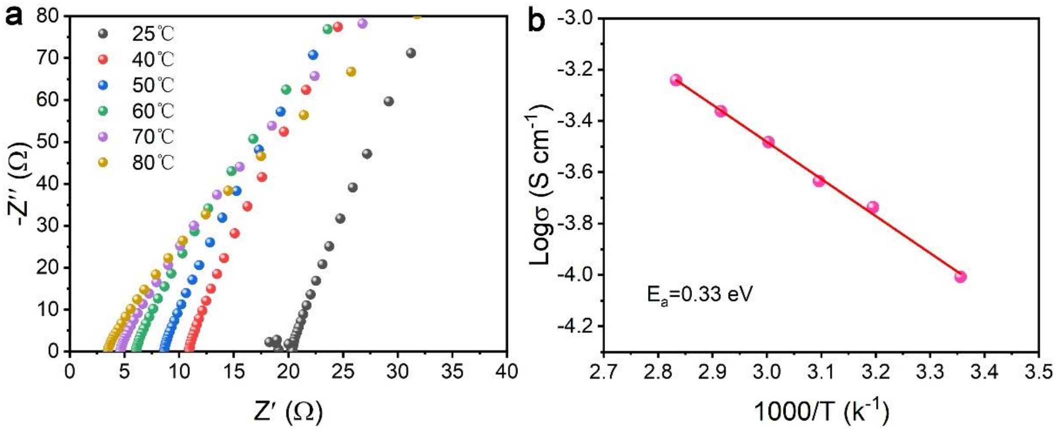

Figure S12. (a) Nyquist plots and the corresponding (b) Arrhenius plots of the control group in which MPC was absent.

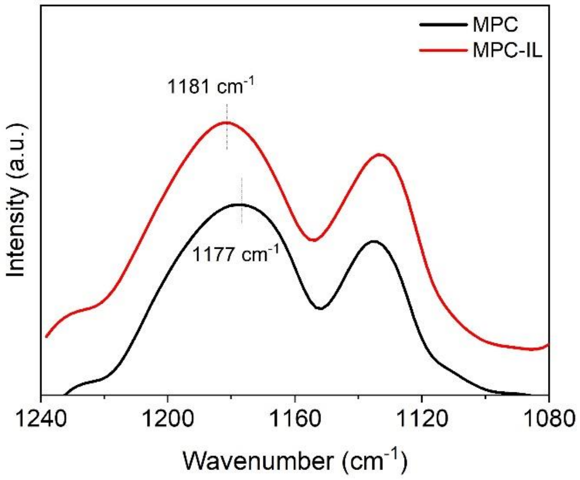

Figure S13. FTIR spectra of MPC and the mixture with IL

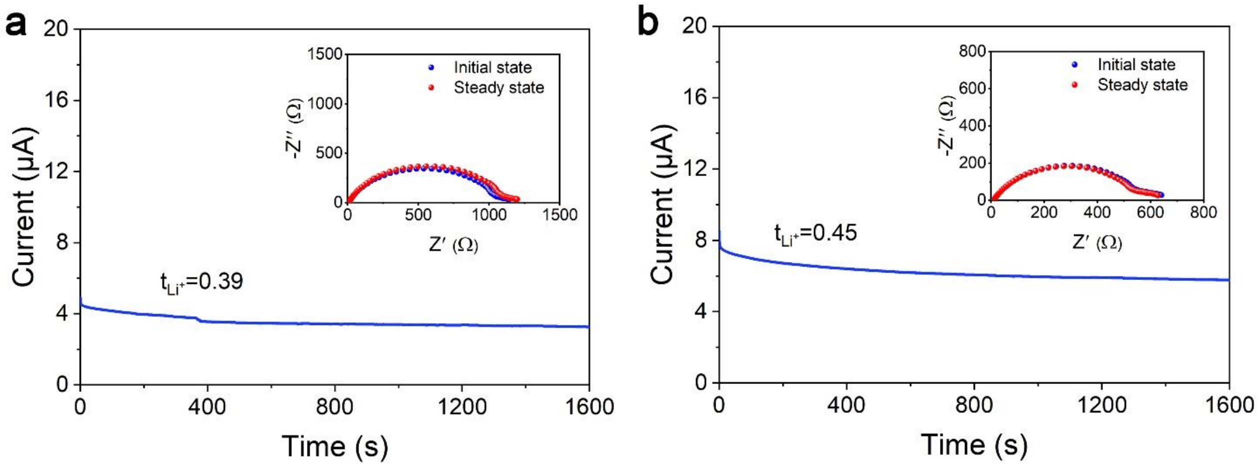

Figure S14. Polarization curve and impedance diagram of the cell before and after polarization (the inset) for (a) Li|GPE-1|Li and (b) Li|GPE-3|Li cell at 25 °C.

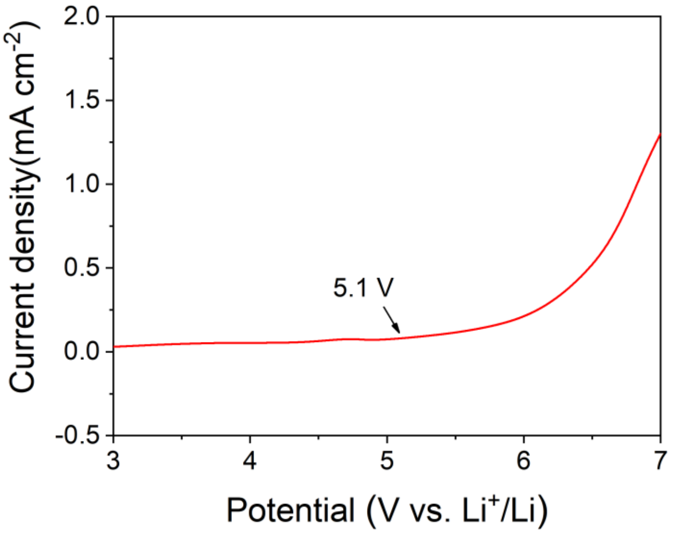

Figure S15. linear sweep voltammetry (LSV) curve of Li|GPE-2|SS.

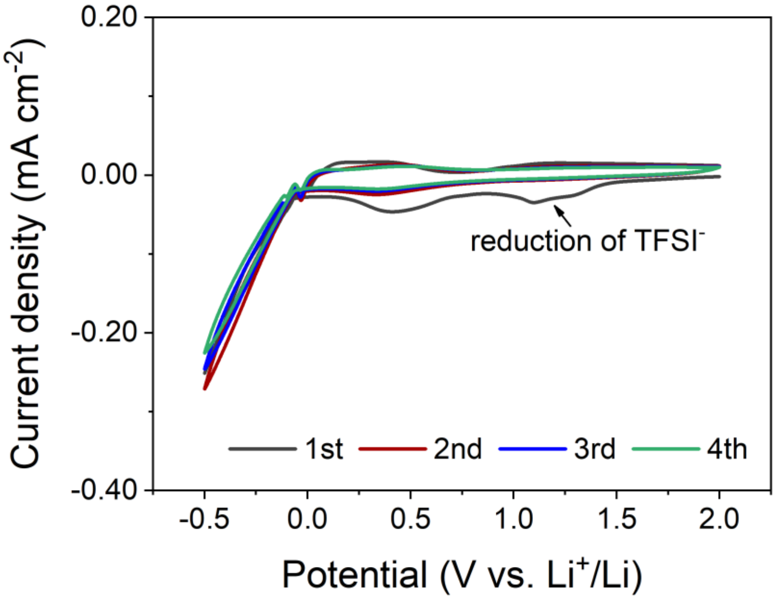

Figure S16. CV curve of the Li|GPE-2 without MPC|SS at a scanning rate of 1 mV s^{-1} at 25 °C.

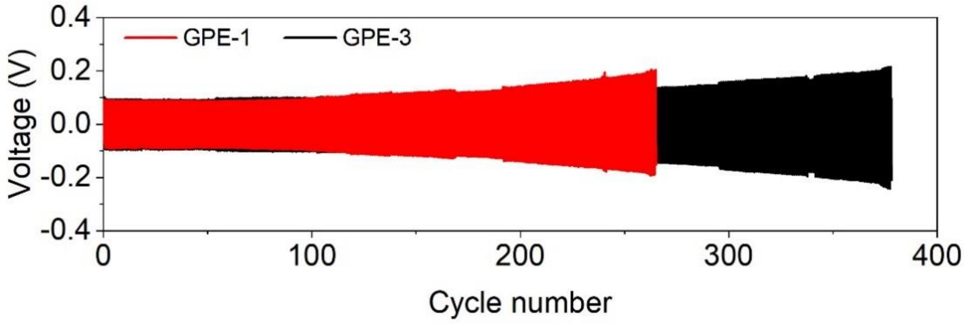

Figure S17. Voltage profiles of Li|GPE-1|Li and Li|GPE-3|Li at current densities of 0.1 mA cm^{-2}**.**

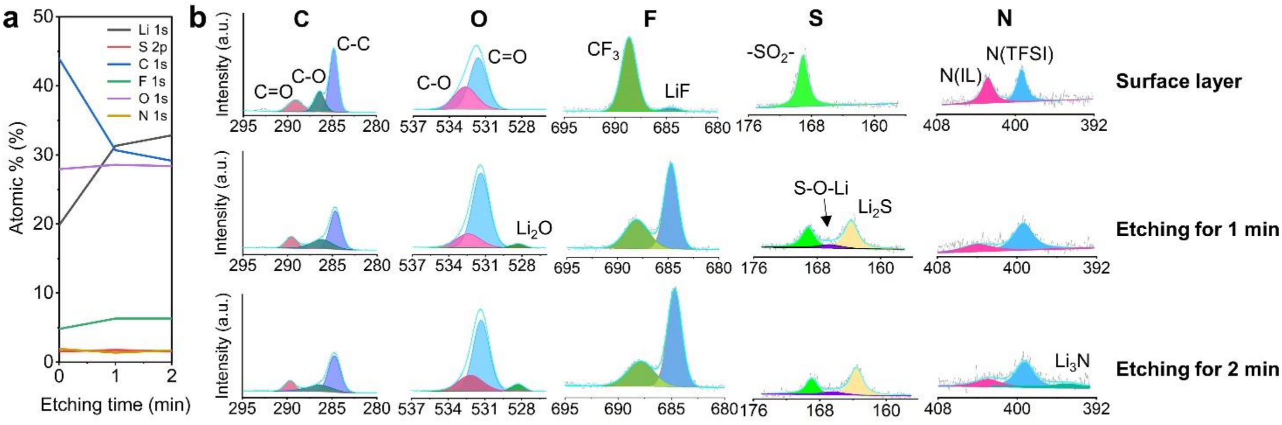

Figure S18. (a) A depth profiling of the elements on the cycled Li anode in Li|GPE-2|Li and the corresponding (b) C, O, F, S and N spectra.

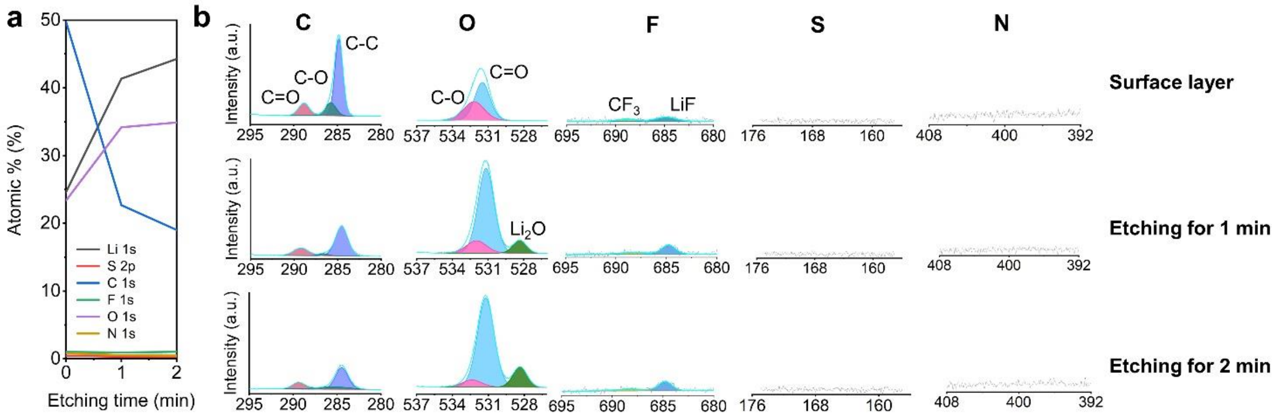

Figure S19. (a) A depth profiling of the elements on the cycled Li anode in Li|GPE-2 without MPC |Li and the corresponding (b) C, O, F, S and N spectra.

ignition test.mp4

Video S1. Ignition test of the fabricated GPE-2 gel electrolyte, which exhibits desirable non-flammability.
